# Impact of environmental pollution, dietary factors and diabetes mellitus on Autism Spectrum Disorder (ASD)

**DOI:** 10.12669/pjms.35.4.269

**Published:** 2019

**Authors:** Abdulrahman Mohammed Alhowikan, Laila Yousef AL-Ayadhi, Dost Muhammad Halepoto

**Affiliations:** 1 Abdulrahman Mohammed Alhowikan, PhD. Department of Physiology, Faculty of Medicine, King Saud University, P O Box 2925, Riyadh 11461 and Saudi Arabia; 2 Laila Yousef AL-Ayadhi, MBBS, PhD. Autism Research and Treatment Center, Department of physiology, Faculty of Medicine, King Saud University, P O Box 2925, Riyadh 11461 and Saudi Arabia; 3 Dost Muhammad Halepoto, PhD. Autism Research and Treatment Center, Department of Physiology, Faculty of Medicine, King Saud University, P O Box 2925, Riyadh 11461 and Saudi Arabia

**Keywords:** Autism Spectrum Disorder, Diabetes Mellitus, Environmental Pollution, Nutritional Factors

## Abstract

Autism spectrum disorder (ASD) is complex neurodevelopmental condition described by impairments in three main behavioral areas: social deficits, impaired communication, and repetitive behaviors. Despite many years of vast study, the causes of ASD are still unknown. Various risk factors including genetic, infectious, metabolic and immunological have been investigated however, environmental, nutritional and diabetes related risk factors have not received sufficient attention. This study has provided an insight into the comprehensive interaction between environmental pollution, dietary factors and diabetes mellitus that could lead to the advancement of this debilitating neurodevelopment disorder. The literature search was done using PubMed and Google Scholar databases up to October 2018. Key words “Environmental Pollution”, “Nutritional Factors”, “Diabetes Mellitus”, “Autism Spectrum Disorder” were selected.

## INTRODUCTION

“Autism spectrum disorders (ASD) are a group of developmental disabilities characterized by deficits in socialization, communication, and repetitive or unusual behaviors”_._^1^ ASD is observed as “multifactorial, with genetic and non-genetic risk factors acting together to produce the phenotype”.^1^ However, the underlying mechanisms still remain to be clarified. There is no genetic or physiological marker reported for autism, the finding is based purely on behavioral, developmental and clinical history. The clinical features of ASD generally appears below age of three years. The disorder affect one in every 70 boys and one in every 310 girls.^2^ Although, the etiopathology of ASD is not well established, the significant research has been dedicated to find out the etiological issues. Although no single reason has been recognized, the existing facts propose that autism results from dissimilar groups of contributory factors comprising genetic, neurobiological and environmental.^3^ The prevalence of Autism Spectrum Disorder (ASD) has increased worldwide dramatically in the past few decades. Studies from the Middle East on this topic have been particularly rare.^4^ Autism in Saudi Arabia is slightly higher than reported in developed countries. One report estimated that in Saudi Arabia there were 42,500 confirmed cases of autism in 2002 and that many more remained undiagnosed.^5^

Previous studies have revealed that “atypical immune activity may play a role in the pathophysiology of ASD”.^6^ Furthermore, the studies allied to autistic patients have confirmed the presence of number of immune dysfunctions.^7^ Earlier studies established that various cytokines and transcription factor activation pathways are considerably elevated in ASD.^8^

### Search Strategies

We searched the databases of PubMed and Google scholar for articles published up until October 2018. Key words “Environmental Pollution”, “Nutritional Factors”, “Diabetes Mellitus”, “Autism Spectrum Disorder” were selected. The manual search of the references of eligible articles for additional studies which were not identified by the electronic search was performed. Only studies published in the English language were included.

***Environmental Factors, Autism And Type 2 Diabetes Mellitus:*** “It has been believed that neuroinflammatory processes and immune dysfunction linked with autism can result following early-life exposure to various environmental pollutants”.^9^ The growing incidence of autism has created major importance in the possible contribution of pollutants in the environment.^10^ Environmental exposure is progressively being identified as possible risk elements for ASD and the probability that the prenatal atmosphere including use of drug, infection, inflammations and contact to liquor and tobacco for the period of gestation may affect the fetal programming during offspring period.^11^ Furthermore, recent research has also established a linked with air pollution, pesticide, heavy metals, toxic waste sites, water pollutants and in-house flooring material to increase the risk of ASD.^12^ A number of studies investigating the contribution of environmental influences in ASD were published.^12^ A substantial volume of studies have been focused on heavy metal, toxicants, phthalates, solvents, pesticides, polychlorinated biphenyls (PCBs) and poly-brominated diphenyl ethers (PBDEs) in relation to ASD.^12^ Several studies have reported the association of heavy metals biomarkers in children with ASD and showed a possible link between toxic environment and autism risk.^12^ Furthermore, direct human exposures including various chemicals, medical trials, nutritional causes or strain involving the parents just before conception, affect the newly born infants. Environmental factors possibly play a role in development of ASD disorders.^13^

Air contamination is a unique risk reason for insulin resistance and prevalence of type 2 diabetes mellitus (T2DM). The association with air pollution and diabetes is greater for traffic related toxins, gaseous, nitrogen dioxide, tobacco smoke and industrial fumes.^14^ Current research suggested that air toxins may contribute to compromised glucose absorption, incidence of insulin resistance and T2DM.^15^ Possible mechanism including oxidative stress and low score inflammation was proposed^16^ which results in loss of insulin signaling^17^ and causes diabetes mellitus.

### Dietary factors, type2 diabetes mellitus (t2dm) and autism

Dietary factors contributing an essential part in supporting good health and a majority of indication has been associated with nutritional deficits to an exacerbation of cognitive function and mental decline.^18^ Though unhealthy diet is usually assumed as a key factor in the progress of T2DM including quantity and varieties of nutritional fat and starches as the risk factors.^19^

Indication from earlier studies shows that dietary factors may play key role in the etio-pathology and management of ASD. An advanced research has also suggested that certain risk factors such as vitamin D and folic acid deficiency in very early age may be linked with autism.^20^ It can be summarized that diet and other environmental impacts might activate an unstable base of genetic tendency, which may lead to the development of autism.^21^ It was observed that irregularities in sugar consumption and absorption could clarify some of the digestive problems in ASD patients, although their role in the neurological and behavioral problems remains unclear.^21^ Nowadays, growing research has highlighted a significant role of bioactive proteins and peptides in ASD. Hodgson et al.^22^ examined the level of redox and methylation metabolites, along with the status of protein homocysteinylation and hair mercury levels, in autistic children. A number of current studies have explained the mechanism of relation between both folic acid and vitamin D and autism.^23^

A recent study reported the role of nutritional factors in the link between autism and small intermissions between pregnancies. It is further suggested that low folate level is related with greater risk of autism.^24^ Numerous assumptions have been suggested and verified to describe the link between vitamin D level of mother and/or autistic child.^23^

Grant and Soles^25^ found that lack of vitamin D in mothers is a possible threating issue for the progress of infantile autism. This may affect fetal brain growth and maternal immune system status in gestation period. Vitamin D can affect various biological methods and performs as an anti-inflammatory agent on brain tissue and exert influences on DNA repair processes.^26^ The importance of Omega-3 fatty acids supplementation received attention and several reports have been suggested as the possible associations between deficiencies of omega-3 fatty acids and behaviors in ASD.^27^ Deficiency of Zn^2+^ has been reported in many autistic children^28^ and found cause for neuropsychological symptoms, learning, and memory impairments.^29^

### Type2 diabetes mellitus (t2dm) and autism

Diabetes mellitus (DM) is a life threatening disease and rapidly growing in all age groups and both sexes. It implicates several physiological roles in various organs and different systems related with extensive and vast health problems.^30^ Association between autism and diabetes mellitus is still not clear, however many factors are suggested to be responsible. Limited research, has examined the possible association between ASD and T2DM. Jones et al.^31^ observed ASD patients from their early to adult age and concluded that ten (10%) of the subjects were diagnosed with T2DM have in their adult life. Several studies have reported that mutual shared genes between ASD and T2DM may increase the possibility of type 2 DM in autistic subjects.^32^ The findings of study by Chen et al.^33^ confirmed that young adults and teenagers with ASD had a greater prevalence of T2DM in future life as compared to without ASD. Furthermore, growing evidence has suggested the possible link between maternal metabolic disorders (obesity and T2DM) and infantile ASD.^34^ These studies proposed that metabolic disorders T2DM, gestational DM and obesity in mothers may elevate the chance of ASD and T2DM in their new born babies.

### Role of Prenatal Diabetes in ASD

“Worldwide diabetes affects up to fifteen percent (15%) of pregnant women.^35^” Gestational diabetes (GDM) result, due to glucose intolerance in beginning of prenatal period,^36^ 7.5% to pre-existing Type-1 diabetes (T1DM), and 5% to pre-existing T2DM.^37^ Li et al.^38^ determined that combination of maternal pre-pregnancy obesity and maternal diabetes were linked with higher possibility for ASD. Moreover, a significant connection between maternal diabetes and risk of ASD in the new born children was confirmed in many studies.^39^

Freeman et al.^40^ found that the incidence of ASD in children with T1DM, may be greater than that in the general population. Doheny^41^ suggested that women with diabetes, hypertension and obesity may have greater chance of having a child with ASD. “For mothers with at least one of these conditions, there was a 60% higher risk for autism in the new born”.

There is indication that mother’s obesity may increase the possibility for deprived neurodevelopmental results in offspring. The link between maternal obesity and compromised mental growth in very young age has been described.^42^ Furthermore, investigation results have also helped that maternal metabolic conditions, including maternal obesity, are linked with higher risk for autism, developmental delay and compromised communication skills.^43^ Furthermore, it has been assumed that inflammation of the fetal brain could be linked to inflammatory processes related with maternal obesity^44^ which may be a potential mechanism for poor neurodevelopmental results detected in newly born babies of mothers with a high body mass index (BMI).

## DISCUSSION

As the prevalence of ASD has increased in the world, it is essential to understand that what factors are contributing in the diet and environment regarding ASD. ASD is likely to start before or after birth affecting the immune system and brain development. The environmental pollution is responsible for alterations in brain growth, as described in reports using thalidomide, valproic acid, and viral infection, in addition to some maternal and paternal factors.^13^

In early fetal growth, brain injury is caused by the exposure to various chemicals, poisonous waste locations, air contaminants and heavy metals.^45^ Several toxic compounds are reported to be responsible for medical neurotoxic effects in adults. The effects of such chemical compounds in the growing human brain are unknown and its effects are not known in children.^45^

Since last ten years, research on environmental risk factors for ASD has been undertaken with significant confirmation that an array of non-genetic factors play a role throughout the pregnancy period and may effect brain development. Numerous studies have reported the significant increase in ASD risk with estimated contact to various risk factors, particularly polluted air, heavy metals during the prenatal period.^46^ Earlier studies have also revealed that genetic and environmental features play a role in the development of autism^3^. Nevertheless, no single neurobiological factor controls the mechanism, pathology and prevalence of autism.^47^

The development of the immune system can be affected by resulting prenatal exposure to toxic pollutant, or incidence of an infection in the early pregnancy period of a developing fetus.^48^ The incidence and contact to toxic substances or infection can rise the occurrence of autoimmune deficits in new born children. However, studies have reported that perinatal inflammation that occurs in the crucial period of fetal growth can result in permanent damage in the cerebral and peripheral areas of the CNS.^9^ Two major sources support the probability of environmental impact for the cause of autism, first the exposure growing human brain to pollutant atmosphere; second generally essential, evidence of concept investigations that especially associate ASD to environmental contacts happening during pergenancy.^48^

Dietary factors play central role in supporting good health, and a majority of evidence has associated with lack of dietary factors for exacerbation of cognitive decline.^18^ Furthermore, dietary associated risk factors such as folic acid, vitamin D, and maternal metabolic syndrome may be responsible for higher incidence in ASD. On the basis of earlier studies, it was assumed that food allergy certainly activates or deteriorate the appearance of neurodevelopmental disorders in children with ASD. Previous studies have indicated that gluten and milk-free diets improved behavior in children with ASD.^49^

Though strong epidemiological indications are restricted and results are provocative, numerous biological mechanisms are suggested in order to explain how DM may cause brain deformity and abnormal neurodevelopment, which supports recent findings.^18^ Li et al, reported^38^ that only the association of obesity and diabetes was linked with main risk of ASD. Developing proof proposes that ASD may be linked to metabolic and immunologic disorders related with diabetes and maternal obesity. In pregnant women obesity increases circulating proinflammatory cytokines^50^ and resulting diabetes encourages proinflammatory environments in intrauterine tissues. Both intrauterine inflammation and fetal brain inflammation are associated in the development of ASD.^51^

Pre gestational diabetes mellitus (PGDM) and gestational diabetes (GDM) are related with many pregnancy problems. PGDM may increase the ratio of inherited abnormalities in the new born children by affecting fetal health and development. GDM, which generally progresses in the 4-6 month of pregnancy, is mostly linked with abnormal fetal development and increased degree of a diversity of problems during prenatal period. PGDM as well as GDM are linked with disturbances in perinatal development and growth, also distressing fine and gross motor growth with learning difficulties and of attention deficit hyperactivity disorder (ADHD) in ASD.^52^ The adverse outcomes of maternal diabetes on the brain may result from intrauterine increased fetal oxidative stress, epigenetic changes in the expression of several genes and additional unidentified reasons. It was described frequently that good management of diabetes during prenatal period may decrease these problems, however not fully prevent them.

There are several possible biological mechanisms,^53^ to clarify the consequence of maternal diabetes, with risk of ASD in offspring. Frist “Maternal hyperglycemia can result in hypoxia in the fetus and a depleted oxygen supply to the fetus may damage neurodevelopment and therefore contribute to a greater risk of ASD”. Second, “maternal hyperglycemia has been associated with increased free-radical production and compromised antioxidant defense system that lead to oxidative stress in the cord blood and placental tissue”. Third, “excessive adiposity that commonly accompanies T2DM and gestational diabetes are inducers of chronic inflammation”. Moreover, it has been assumed that insulin signaling may share to advancement of ASD in hereditarily vulnerable subjects via triggering of PI3K/T or pathway in neurons. Fourth, “T1DM is an autoimmune disorder resulting from a cellular mediated autoimmune destruction of pancreatic beta-cells”. Maternal modified autoimmunity may effect brain growth in the newly born children by forming a hostile intrauterine atmosphere or by modifying the young children’s autoimmunity in initial growth through immunoglobulin G. Fifth, “epigenetic modification by hyperglycemia may also be implicated in the pathogenesis of ASD.”

## CONCLUSIONS

“Autism is a biologically based neurodevelopmental disorder of brain”. Environmental and nutritional factors may play an important role in determining the risk of ASD. This review highlights various environmental and nutritional factors which appear to be linked with greater risk for autism. However key risk factors are still needed to be recognized. Current literature review suggested that the etiology of ASD may involve complex connections between environmental and nutritional factors that may perform synergistically during acute phases of neurodevelopment in an approach that increases the chance to have diabetes in ASD. Diabetes mellitus is linked with a significantly increased risk of ASD. However, the quantity of existing literature is still limited. Furthermore, to understand the association in better way, more studies discovering the core molecular mechanisms are required. Additional studies may also be necessary to explain the biological association between ASD and T2 DM.
